# Comparison of perioperative complications and health‐related quality of life between robot‐assisted and open radical cystectomy: A systematic review and meta‐analysis

**DOI:** 10.1111/iju.14005

**Published:** 2019-05-13

**Authors:** Shoji Kimura, Takehiro Iwata, Beat Foerster, Nicola Fossati, Alberto Briganti, Yasutomo Nasu, Shin Egawa, Mohammad Abufaraj, Shahrokh F Shariat

**Affiliations:** ^1^ Department of Urology Medical University of Vienna Vienna Austria; ^2^ Department of Urology Jikei University School of Medicine Tokyo Japan; ^3^ Department of Urology Okayama University Graduate School of Medicine Dentistry and Pharmaceutical Sciences Okayama Japan; ^4^ Department of Urology Kantonsspital Winterthur Winterthur Switzerland; ^5^ Unit of Urology Urological Research Institute IRCCS San Raffaele Scientific Institute Milan Italy; ^6^ Vita‐Salute San Raffaele University Milan Italy; ^7^ Department of Special Surgery Jordan University Hospital The University of Jordan Amman Jordan; ^8^ Department of Urology Weill Cornell Medical College New York New York USA; ^9^ Department of Urology University of Texas Southwestern Medical Center Dallas Texas USA; ^10^ Karl Landsteiner Institute of Urology and Andrology Vienna Austria; ^11^ Institute for Urology and Reproductive Health Sechenov University Moscow Russia

**Keywords:** health‐related quality of life, meta‐analysis, open radical cystectomy, postoperative complication, robot‐assisted radical cystectomy

## Abstract

To compare postoperative complications and health‐related quality of life of patients undergoing robot‐assisted radical cystectomy with those of patients undergoing open radical cystectomy. A systematic search was carried out according to the Preferred Reporting Items for Systematic Reviews and Meta‐Analyses statement. A pooled meta‐analysis was carried out to assess the differences between robot‐assisted radical cystectomy and open radical cystectomy according to randomized and non‐randomized comparative studies, respectively. We identified six randomized comparative studies and 31 non‐randomized comparative studies. Most robot‐assisted radical cystectomy patients were treated with extracorporeal urinary diversion. Robot‐assisted radical cystectomy was associated with longer operative times, and lower blood loss and transfusion rates compared with open radical cystectomy in both randomized comparative studies and non‐randomized comparative studies. There was no significant difference between robot‐assisted radical cystectomy and open radical cystectomy in the rate of patients with any or major complications within 90 days both in randomized comparative studies and non‐randomized comparative studies. Non‐randomized comparative studies reported a lower rate of complications at 30 days, mortality at 90 days and length of stay for patients treated with robot‐assisted radical cystectomy, which were not confirmed in randomized comparative studies. Additionally, there were no differences in postoperative quality of life score assessment at 3 and 6 months between robot‐assisted radical cystectomy and open radical cystectomy. Robot‐assisted radical cystectomy is associated with less blood loss and lower transfusion rates. There is no difference in complications, length of stay, mortality, and quality of life between robot‐assisted radical cystectomy and open radical cystectomy. Data from non‐randomized comparative studies favor perioperative outcomes in robot‐assisted radical cystectomy patients, the failure to confirm in randomized comparative studies, likely due to bias in study design and reporting. Further randomized comparative studies comparing postoperative complications and quality of life between robot‐assisted radical cystectomy with intracorporeal urinary diversion and open radical cystectomy are required to assess potential differences between these two surgical approaches.

**Abbreviations & Acronyms**
ASA: American Society of Anesthesiologists
BCI: Bladder Cancer Index urinary/sexual/bowel
BIS: body image scale
BMI: body mass index
CARE: Convalescence and Recovery Evaluation
CI: confidence interval
ECUD: extracorporeal urinary diversion
ERAS: enhanced recovery after surgery
FACT‐VCI: Functional Assessment of Cancer Therapy‐Vanderbilt Cystectomy Index
ICUD: intracorporeal urinary diversion
M–H: Mantel–Haenszel test
NR: not reported
NRCT: non‐randomized comparative study
ORC: open radical cystectomy
PRISMA: Preferred Reporting Items for Systematic Reviews and Meta‐Analyses
QLQ‐C30: Quality of Life Questionnaire Core 30
QOL: quality of life
RARC: robot‐assisted radical cystectomy
RC: radical cystectomy
RCTs: randomized controlled studies
ROBINS‐I: risk of bias in non‐randomized studies of interventions
RR: relative risk
SD: standard deviation
UTI: urinary tract infection
WMD: weighted mean difference

## Introduction

ORC with pelvic lymph node dissection and urinary diversion is the standard treatment for patients with muscle‐invasive bladder cancer and those with very high‐risk non‐muscle‐invasive bladder cancer.^1^ ORC continues to be associated with a high rate of mortality and morbidity, such as perioperative UTIs, thrombosis and ileus, which cause prolonged hospital stay and affect the patients’ health‐related QOL. Indeed, >60% of patients treated with ORC experience at least one perioperative complication, and approximately 20% experience a high‐grade complication within 90 days of the surgery.^2^ Although progress has resulted from changes in perioperative management, such as the ERAS, a proposed strategy to reduce perioperative complications has been to minimize the invasiveness of the surgical procedures, such as through the performance of a RARC.

Initial non‐randomized retrospective studies showed significant advantages to RARC over ORC, such as lower estimated blood loss, lower blood transfusion rates, shorter length of stay and lower rate of postoperative complications.^3^ Conversely, early randomized controlled trials failed to find any differences in complications at 90 days between RARC and ORC.^4^, ^5^ As the last meta‐analysis on this subject was carried out in 2017,^6^ a significant body of novel data including both randomized (RCTs) and NRCTs have emerged, adding new evidence to the topic.^2^, ^5^, ^7^, ^8^

To assess the differential perioperative complications and health‐related QOL outcomes between RARC and ORC, we carried out an up‐to‐date systematic review and meta‐analysis of the literature comparing complications rates, as well as health‐related QOL outcomes of patients treated with RARC, with those of patients treated with ORC. We analyzed the data from the RCTs and the NRCTs separately to unmask potential bias arising from study design.

## Methods

The protocol has been registered in the International Prospective Register of Systematic Reviews database (PROSPERO: CRD42018108001). The PRISMA checklist is reported in Table S1.

### Literature search and inclusion/exclusion criteria

The present systematic review and meta‐analysis were carried out according to the PRISMA statement^9^ and the Cochrane Handbook for Systematic Reviews of Interventions.^10^ A comprehensive literature search using the electronic databases (MEDLINE, Web of Science, Scopus and Cochrane Library) was carried out on 10 August 2018 to retrieve the articles published comparing postoperative complications and health‐related QOL of patients treated with RARC with that of those treated with ORC. All full text papers were assessed and excluded with reasons when deemed inappropriate after screening based on the study title and abstract. Two reviewers carried out this process independently. Disagreements were resolved by a third party. The following string terms were used: (bladder cancer) AND (postoperative complication OR intraoperative complication OR perioperative complication OR postoperative morbidity OR postoperative mortality OR quality of life OR length of stay) AND (robot‐assisted radical cystectomy OR da Vinci radical cystectomy OR robotic radical cystectomy*)*.

Studies were included if they compared RARC with ORC and reported at least one postoperative complication outcome or health‐related QOL assessment between both arms in RCTs or NRCTs that included retrospective observational or cohort studies. If there were multiple articles written by the same group based on a similar patient cohort, only the largest or most recently published study was included. Review articles, editorials, comments and meeting abstracts were excluded. References of included manuscripts were scanned for additional studies of interest.

### Data extraction

Two authors independently extracted the required data. The baseline patient characteristics, postoperative complication rate, and health‐related QOL assessment data of both RARC and ORC arms in all eligible studies were collected. The outcomes of interest in this meta‐analysis were postoperative complication rates within 30 and 90 days, types of complications, and other perioperative parameters, such as operative time, estimated blood loss, blood transfusion rate and length of hospital stay, as well as health‐related QOL scores. All discrepancies regarding data extraction were resolved by consensus or finally decided by Delphi consensus with other authors.

### Statistical analysis

The WMD and RR with 95% CIs were used as the summary variables for continuous and dichotomous outcomes, respectively. If studies only reported continuous data as the median and range or interquartile range, the mean and SDs were calculated according to Hozo *et al*.^11^ Statistical heterogeneity among studies was calculated using the *I* ^2^ statistics. The χ^2^‐test and *I* ^2^ statistics with significance set at *P* <0.10 and *I* ^2^ <50%, respectively, were used to assess statistical heterogeneity among studies. If there was a lack of heterogeneity, fixed effects models were used for meta‐analysis. Random effects models were used in cases of heterogeneity. Statistical analyses were carried out using Review Manager (RevMan‐Computer program, Version 5.3 Copenhagen: The Nordic Cochrane Centre, The Cochrane Collaboration, 2014).

### Risk of bias

For RCTs, we used the Cochrane Risk of Bias Tool for RCTs.^10^ For NRCTs, we used the ROBINS‐I tool.^12^ Two authors independently assessed the risk of bias in each study. All discrepancies regarding the risk of bias were resolved by consensus between two authors.

## Results

### Study selection and characteristics

**Figure 1:**
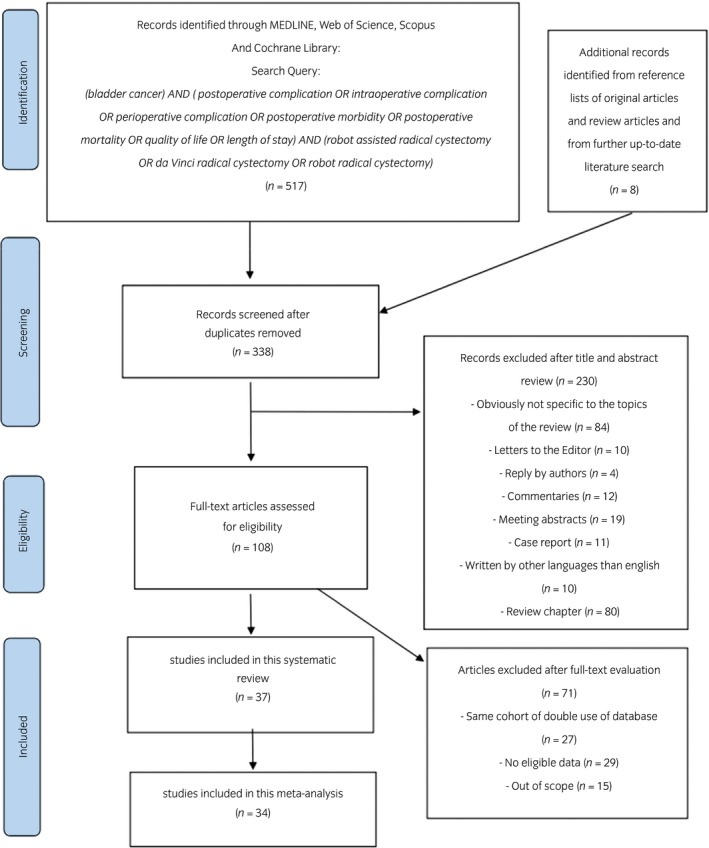
PRISMA flow chart of the systematic review and meta‐analysis.

Overall, 525 articles were identified for an initial assessment (Fig. 1); 187 duplicates were removed. Then, 230 and 71 articles were excluded after title and abstract assessment, and full text reading, respectively. Finally, 37 and 34 studies that reported postoperative complications and health‐related QOL outcomes between RARC and ORC were included for qualitative and quantitative analyses, respectively. The general characteristics of the eligible studies are summarized in Table 1. The present systematic review includes six RCTs^2^, ^4^, ^5^, ^13^, ^14^, ^15^ comprising 581 patients, and 31 NRCTs^7^, ^8^, ^16^, ^17^, ^18^, ^19^, ^20^, ^21^, ^22^, ^23^, ^24^, ^25^, ^26^, ^27^, ^28^, ^29^, ^30^, ^31^, ^32^, ^33^, ^34^, ^35^, ^36^, ^37^, ^38^, ^39^, ^40^, ^41^, ^42^, ^43^, ^44^ comprising 48 392 patients published between 2010 and 2018. Of these, 20 studies enrolled patients from North America, nine from Europe and eight from Asia. Four RCTs and 19 NRCTs had only patients treated with ECUD. Just five NRCTs included patients treated with ICUD. Other studies included mix cohorts with ECUD/ICUD or did not report on the type of urinary diversion. Three studies compared the complication rates between RARC and ORC only in patients who received a neobladder. Perioperative parameters are shown in Table 2. Operative time and length of stay were reported in 31 and 30 studies, respectively. Estimated blood loss and transfusion rate were reported in 31 and 20 studies, respectively. Postoperative complications after RARC and ORC are shown in Table 3; 27 studies investigated complication rates within 30 or 90 days. The mortality rate within 90 days was reported in 15 studies. Postoperative health‐related QOL is shown in Table 4. Just three RCTs and three NRCTs reported comparisons of health‐related QOL changes before and after RARC and ORC. It was not possible to carry out a meta‐analysis of the health‐related QOL outcomes due to the heterogeneity of health‐related QOL assessment tools between included studies. The risk of bias tables in RCTs and NRCTs are shown in Figure S1 and Table S2, respectively.

**Table 1:** Characteristics of the included studies

Author	Region	Arm	No. patients	Age, years (mean or median)	BMI (mean or median)	ASA score (mean or median)	Urinary diversion (corporeal)	Rate of neobladder (%)
Randomized study								
Nix 2010	USA	RARC/ORC	21/20	67.4/69.2	27.5/28.4	2.71/2.70	ECUD	33.3/30
Parekh 2013	USA	RARC/ORC	20/20	69.5/64.5	27.6/28.3	3.0/3.0	NR	NR
Messer 2014	USA	RARC/ORC	20/20	69.5/64.5	27.6/28.3	3.0/3.0	NR	NR
Bochner 2015	USA	RARC/ORC	60/58	66/65	27.9/29.0	2.73/2.73	ECUD	55/55
Khan 2016	UK	RARC/ORC	20/20	68.6/66.6	27.5/27.4	1.85/1.85	ECUD	10/15
Parekh 2018	USA	RARC/ORC	150/152	70/67	27.8/28.2	NR	ECUD	24/20
Non‐randomized study								
Ng 2010	USA	RARC/ORC	83/104	70.9/67.2	26.3/27.2	43.3/48.1 (%, 3≤)	ECUD	31.3/27.9
Richard 2010	USA	RARC/ORC	35/35	65/66	27/26	89/77 (%, 3≤)	ECUD	8.6/0
Gondo 2012	Japan	RARC/ORC	11/15	68.9/69.7	21.8/24.2	NR	ECUD	36.4/40.0
Khan 2012	UK	RARC/ORC	48/52	66.5/65	NR	18.8/48.1 (%, 3≤)	ECUD	12.5/9.6
Styn 2012	USA	RARC/ORC	50/100	66.6/65.6	29.8/29.6	54/57 (%, 3≤)	ECUD	28/28
Sung 2012	Korea	RARC/ORC	35/104	62.2/65.9	23.1/22.4	8.6/7.7 (%, 3≤)	ECUD	62.9/18.3
Kander 2013	USA	RARC/ORC	100/100	67/67	26.5/27.1	78/73 (%, 3≤)	ECUD	3.0/12.0
Knox 2013	USA	RARC/ORC	58/84	65.9/67.1	28.6/28.9	88/90 (%, 3≤)	ECUD	8.6/10.7
Maes 2013	USA	RARC/ORC	14/14	71.0/67.6	27.3/27.2	NR	ECUD	0/0
Aboumohamed 2014	USA	RARC/ORC	82/100	71.5/71.5	27.8/27.9	45.1/66.0 (%, 3≤)	ICUD/ECUD	0/0
Leow 2014	USA	RARC/ORC	2667/40 980	NR	NR	NR	NR	8.5/6.1
Musch 2014	Germany	RARC/ORC	100/42	71.4/69.0	27/27	NR	ECUD	22.0/16.7
Niegisch 2014	Germany	RARC/ORC	64/79	68/71	24/26	2.0/3.0	ECUD	34.3/32.9
Koupparis 2015	UK	RARC/ORC	102/56	68.2/66.4	NR	26.5/8.9 (%, 3≤)	ICUD	10.8/7.1
Bak 2016	Korea	RARC/ORC	42/70	70/70	22.2/22.3	2.0/2.0	ECUD	26.2/1.4
Cusano 2016	USA	RARC/ORC	121/92	65.9/67.8	28.2/28.4	3.0/3.0	ICUD/ECUD	30.0/25.0
Gandaglia 2016	Belgium	RARC/ORC	138/230	70/70.9	26.1/26.0	39.1/38.7 (%, 3≤)	ICUD/ECUD	15.2/62.5
Iwamoto 2016	Japan	RARC/ORC	20/40	73/72.5	23.1/21.7	NR	ECUD	15.0/22.5
Li 2016	USA	RARC/ORC	57/267	67.0/65.7	NR	NR	NR	NR
Satkunasivam 2016	USA	RARC/ORC	28/79	63.5/67	NR	60.7/71.8 (%, 3≤)	ICUD	100/100
Winters 2016	USA	RARC/ORC	29/58	79.2/79.6	26.9/26.5	NR	ECUD	NR
Kingo 2017	Denmark	RARC/ORC	38/125	68.3/72.3	27.3/26.4	5.3/23.2 (%, 3≤)	ICUD	100/100
Koie 2017	Japan	RARC/ORC	29/196	65/69	NR	NR	NR	NR
Muto 2017	Japan	RARC/ORC	21/28	66.8/70.3	23.7/23.1	NR	ECUD	28.6/3.6
Sharma 2017	USA	RARC/ORC	65/407	70.9/70.2	27.8/28.0	49.2/56.0 (%, 3≤)	ECUD	10.8/13.8
Flamiatos 2018	USA	RARC/ORC	100/149	NR	27.8/28.2	NR	ECUD	16.0/24.8
Kukreja 2018	USA	RARC/ORC	100/96	66.2/66.2	28.9/29.4	NR	NR	NR
Simone 2018	Italy	RARC/ORC	64/46	62.5/63.6	26.1/26.7	12.5/13.0 (%, 3≤)	ICUD	100/100
Tan 2018	UK	RARC/ORC	50/45	62.8/65.0	27.0/29.7	38.0/13.3 (%, 3≤)	ICUD	14.0/15.6
Panwar 2018	India	RARC/ORC	24/54	57/58	23.2/23.1	NR	ECUD	33.3/11.1
Ram 2018	India	RARC/ORC	125/45	61.8/60.1	24.2/23.9	NR	ECUD	36.0/28.9

**Table 2:** Perioperative parameters after RARC and ORC

Author	Arms	Operation time, min (mean or median)	EBL, mL (mean or median)	Transfusion rate (%)	LOS, days (mean or median)
Randomized studies					
Nix 2010	RARC/ORC	252/211.2	258/575	NR	5.1/6.0
Parekh 2013	RARC/ORC	300/285.5	400/800	40/50	6.0/6.0
Messer 2014	RARC/ORC	300/385.5	400/800	40/50	6.0/6.0
Bochner 2015	RARC/ORC	456/329	516/676	NR	8.0/8.0
Khan 2016	RARC/ORC	389/293	585/808	NR	11.9/14.4
Parekh 2018	RARC/ORC	428/361	300/700	24/45	6.0/7.0
Non‐randomized studies					
Ng 2010	RARC/ORC	375/357	460/1172	NR	8.0/5.5
Richards 2010	RARC/ORC	530/420	350/1000	17.1/71.4	7.0/8.0
Gondo 2012	RARC/ORC	409/364	657/1789	0/40.0	40.2/37.0
Khan 2012	RARC/ORC	440/372	381/1407	4.2/57.7	9.8/19.3
Styn 2012	RARC/ORC	455/349	350/475	4/24	9.5/10.2
Sung 2012	RARC/ORC	578/501	448/1063	11.4/56.7	28.9/27.1
Kader 2013	RARC/ORC	451/393	423/986	15/47	6.0/8.0
Knox 2013	RARC/ORC	468/396	276/1522	5.2/80.1	6.3/10.8
Maes 2013	RARC/ORC	383/268	350/800	7.1/28.6	12/11.4
Aboumohamed 2014	RARC/ORC	382/250	444/489	NR	8.0/7.0
Leow 2014	RARC/ORC	386/338	NR	NR	10.2/11.8
Musch 2014	RARC/ORC	404/333	300/600	27.0/59.5	14.0/15.5
Niegisch 2014	RARC/ORC	360/360	300/800	NR	13.0/16.0
Koupparis 2015	RARC/ORC	NR	NR	19.6/41.1	NR
Bak 2016	RARC/ORC	480/405	300/598	23.8/45.7	19.0/21.0
Cusano 2016	RARC/ORC	508/403	450/600	21.5/39.1	8.0/9.0
Gandaglia 2016	RARC/ORC	330/185	300/300	9.4/16.1	13.0/20.0
Iwamoto 2016	RARC/ORC	512/523	227/935	10.0/92.5	NR
Winters 2016	RARC/ORC	413/370	257/641	10.3/32.8	7.0/9.0
Kingo 2017	RARC/ORC	311/272	185/1823	NR	10.9/9.2
Koie 2017	RARC/ORC	496/269	330/1150	NR	NR
Muto 2017	RARC/ORC	561/493	458/1235	NR	19.0/25.5
Sharma 2017	RARC/ORC	423/302	350/800	NR	6/7
Flamiatos 2018	RARC/ORC	411/371	150/600	NR	5.0/7.0
Kukreja 2018	RARC/ORC	NR	640/1158	12.0/39.6	NR
Simone 2018	RARC/ORC	NR	NR	9.4/93.5	NR
Tan 2018	RARC/ORC	NR	NR	NR	13.6/20.1
Panwar 2018	RARC/ORC	452/351	552/512	NR	22.9/23.5
Ram 2018	RARC/ORC	364/355	229/122	NR	10.2/12.4

**Table 3:** Postoperative complications after RARC and ORC

Author	Arm	Complication within 30 days (Clavien 1–5) (%)	Complication within 30 days (Clavien 3≤) (%)	Complication within 90 days (Clavien 1–5) (%)	Complication within 90 days (Clavien 3≤) (%)	Mortality rate within 90 days (%)	UTI rate (%)	Thrombosis rate (%)	Ileus rate (%)
Randomized studies									
Nix 2010	RARC/ORC	33/50	NR	NR	NR	NR	9.5/5	4.8/0	9.5/15
Parekh 2013	RARC/ORC	25/25	NR	NR	NR	NR	NR	NR	5/5
Messer 2014	RARC/ORC	NR	NR	NR	NR	NR	NR	NR	NR
Bochner 2015	RARC/ORC	NR	NR	NR	21.7/20.7	0/1.7	NR	8.3/8.6	NR
Khan 2016	RARC/ORC	55/70	30/20	55/70	35/20	NR	35/15	5/0	5/35
Parekh 2018	RARC/ORC	NR	NR	67.3/69.1	22/22.4	2.7/2.6	35.3/25.7	4.7/7.9	22/20.4
Non‐randomized studies									
Ng 2010	RARC/ORC	41.0/58.7	9.6/29.8	41.0/58.7	16.9/30.8	0/5.8	16.9/16.3	6.0/3.8	15.7/20.2
Richards 2010	RARC/ORC	60.0/65.7	20.0/25.7	NR	NR	NR	NR	NR	NR
Gondo 2012	RARC/ORC	54.5/73.3	0/6.7	NR	NR	NR	NR	NR	NR
Khan 2012	RARC/ORC	NR	NR	41.7/71.2	16.7/28.8	0/1.9	2.1/5.8	0/5.8	8.3/13.5
Styn 2012	RARC/ORC	66.0/62.0	NR	NR	NR	0/3.0	NR	NR	22/25
Sung 2012	RARC/ORC	NR	NR	62.9/74.0	8.6/23.1	2.9/2.9	20/7.7	0/0	25.7/29.8
Kader 2013	RARC/ORC	NR	NR	36.0/58.0	10/22	1.0/0	NR	NR	NR
Knox 2013	RARC/ORC	43.1/64.3	25.9/22.6	NR	NR	NR	1.7/6.0	1.7/3.6	10.3/7.1
Maes 2013	RARC/ORC	NR	NR	NR	NR	NR	NR	NR	57/58
Aboumohamed 2014	RARC/ORC	NR	NR	NR	23.2/16.0	NR	NR	NR	NR
Leow 2014	RARC/ORC	NR	NR	59.7/56.8	19.8/17.0	2.5/3.8	NR	NR	NR
Musch 2014	RARC/ORC	NR	NR	NR	NR	2.0/4.8	29.0/28.6	3.0/19.0	8.0/38.1
Niegisch 2014	RARC/ORC	NR	NR	NR	NR	3.1/3.8	NR	NR	NR
Koupparis 2015	RARC/ORC	NR	NR	NR	NR	2.0/1.8	NR	NR	NR
Bak 2016	RARC/ORC	NR	NR	NR	NR	NR	21.4/20.0	0/0	21.4/22.9
Cusano 2016	RARC/ORC	NR	NR	47.1/54.3	18.2/20.7	NR	NR	NR	NR
Gandaglia 2016	RARC/ORC	NR	NR	60.1/44.3	15.9/20.4	1.4/0.9	NR	NR	NR
Iwamoto 2016	RARC/ORC	100/100	5.0/10.0	NR	NR	NR	NR	NR	0/12.5
Winters 2016	RARC/ORC	NR	NR	37.9/37.9	NR	NR	NR	NR	NR
Kingo 2017	RARC/ORC	NR	26.3/17.6	NR	31.6/23.2	NR	21.1/9.6	0/4.8	13.1/9.6
Koie 2017	RARC/ORC	NR	0/0	NR	0/0	0/0	0/0	0/0	17.2/3.8
Muto 2017	RARC/ORC	NR	NR	NR	NR	NR	9.5/10.7	0/3.6	4.8/3.6
Sharma 2017	RARC/ORC	52.3/59.7	13.8/19.7	NR	NR	NR	NR	NR	NR
Flamiatos 2018	RARC/ORC	66.0/85.9	10.0/19.5	NR	NR	1.0/2.0	NR	0/0.7	NR
Kukreja 2018	RARC/ORC	NR	NR	48.0/46.9	12.0/9.4	NR	NR	NR	NR
Simone 2018	RARC/ORC	39.1/45.7	6.3/2.2	NR	NR	NR	NR	NR	NR
Tan 2018	RARC/ORC	64.0/71.1	22.0/23.9	78.0/86.0	26.0/30.2	6.0/7.0	NR	NR	47.5/31.1
Panwar 2018	RARC/ORC	NR	NR	NR	NR	NR	0/0	0/0	4.2/18.5
Ram 2018	RARC/ORC	39.2/55.6	15.2/22.2	NR	NR	NR	0/0	0/0	9.6/11.1

**Table 4:** Postoperative health‐related QOL change

Author	QOL scale	Arm	QOL score baseline	QOL score at 1 month	QOL score at 3 months	QOL score at 6 months	QOL score at 9 months	QOL score at 12 months	QOL score overall
Randomized study									
Messer 2014	FACT‐VCI	RARC/ORC	119/135	NR	126.5/135.5 *P* = 0.85	121.5/126 *P* = 0.58	141.5/127.5 *P* = 0.63	116/129 *P* = 0.48	NR
Bochner 2015	QLQ‐C3 (global QOL)	RARC/ORC	78/75	NR	77/72 *P* = 0.4	76/78 *P* = 0.5	NR	NR	NR
Parekh 2018	FACT‐VCI	RARC/ORC	120.1/120.9	NR	122.8/125.2	126.0/127.5	NR	NR	NR
Non‐randomized study									
Aboumohamed 2014	BCI (urinary/sexual/bowel)	RARC ORC	76.2/44.1/83.0 78.9/48.5/83.7	77.9/23.8/73.0 72.9/28.8/74.1	79.0/22.5/79.7 79.2/40.5/83.4 –/*P* = 0.08/–	81.2/28.7/80.4 84.6/35.3/26.3	NR	84.8/28.9/83.2 85.2/36.8/85.4	*P* = 0.11/*P* = 0.047/*P* = 0.58
	BIS	RARC ORC	2.1 2.9	4.8 4.9	5.8 4.8	6.6 3.9 *P* = 0.24	NR	6.2 5.6	*P* = 0.93
Li 2016	BCI (urinary/sexual/bowel)	RARC/ORC	NR	NR	NR	NR	NR	NR	*P* = 0.63/*P* = 0.37/*P* = 0.72
Satkunasivam 2016	BCI (urinary)	RARC/ORC	NR	NR	NR	NR	NR	NR	69.5/73.7/*P* = 0.44

### Meta‐analysis

#### Comparison of perioperative parameters between RARC and ORC

**Figure 2:**
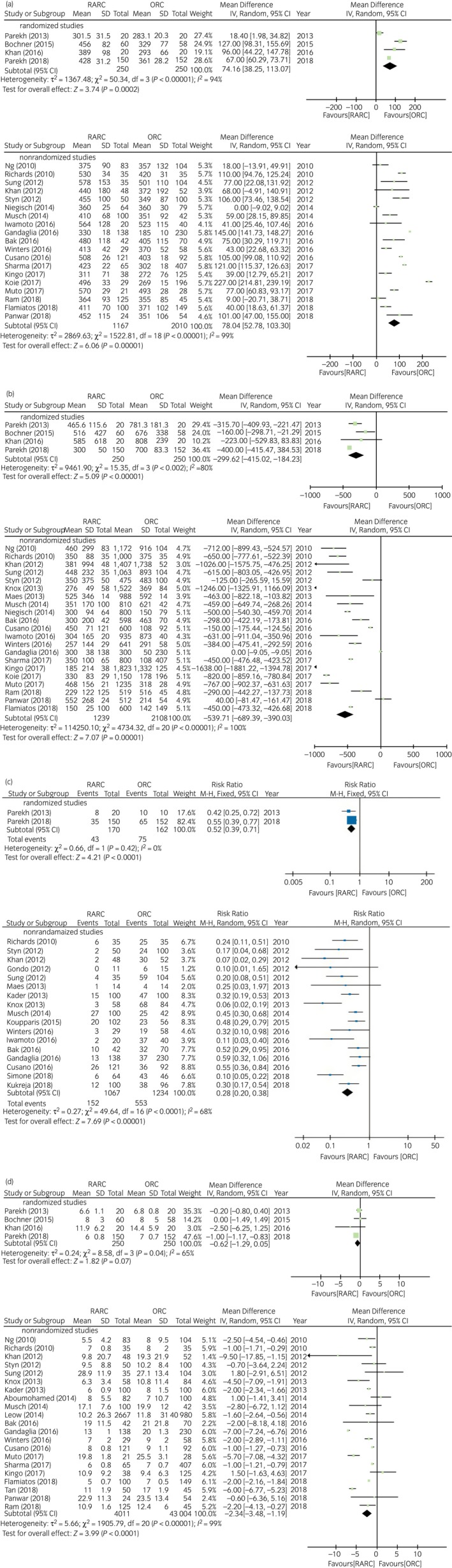
Forest plots showing the comparison of (a) operative time, (b) blood loss, (c) transfusion rate and (d) length of stay between RARC and ORC.

The forest plots (Fig. 2a) showed that RARC was associated with longer operative times in both RCTs (WMD: 74.16 min, 95% CI 35.25–113.07, *P* = 0.0002) and NRCTs (WMD: 78.04 min, 95% CI 52.78–103, *P* < 0.00001). Figure 2b,c show that RARC was associated with a reduction in estimated blood loss in both RCTs (WMD: −299.62 mL, 95% CI −415.02 to −184.23, *P* < 0.00001) and NRCTs (WMD: −539.71 mL, 95% CI −689.39 to −390.03, *P* < 0.00001), as well as lower transfusion rates in RCTs (RR 0.52, 95% CI 0.39–0.71, *P* < 0.0001) and NRCTs (RR 0.28, 95% CI 0.20–0.38, *P* < 0.00001). RARC was associated with shorter length of stay in NRCTs (WMD: −2.34, 95% CI −3.48 to −1.19, *P* < 0.0001), but not in RCTs (WMD: −0.62 days, 95% CI −1.29 to 0.05, *P* = 0.07; Fig. 2d). The χ^2^‐test and *I* ^2^‐test showed heterogeneity, except in pooled analysis of the transfusion rate in RCTs (Fig. 2c).

#### Comparison of postoperative complications within 30 and 90 days between RARC and ORC

**Figure 3:**
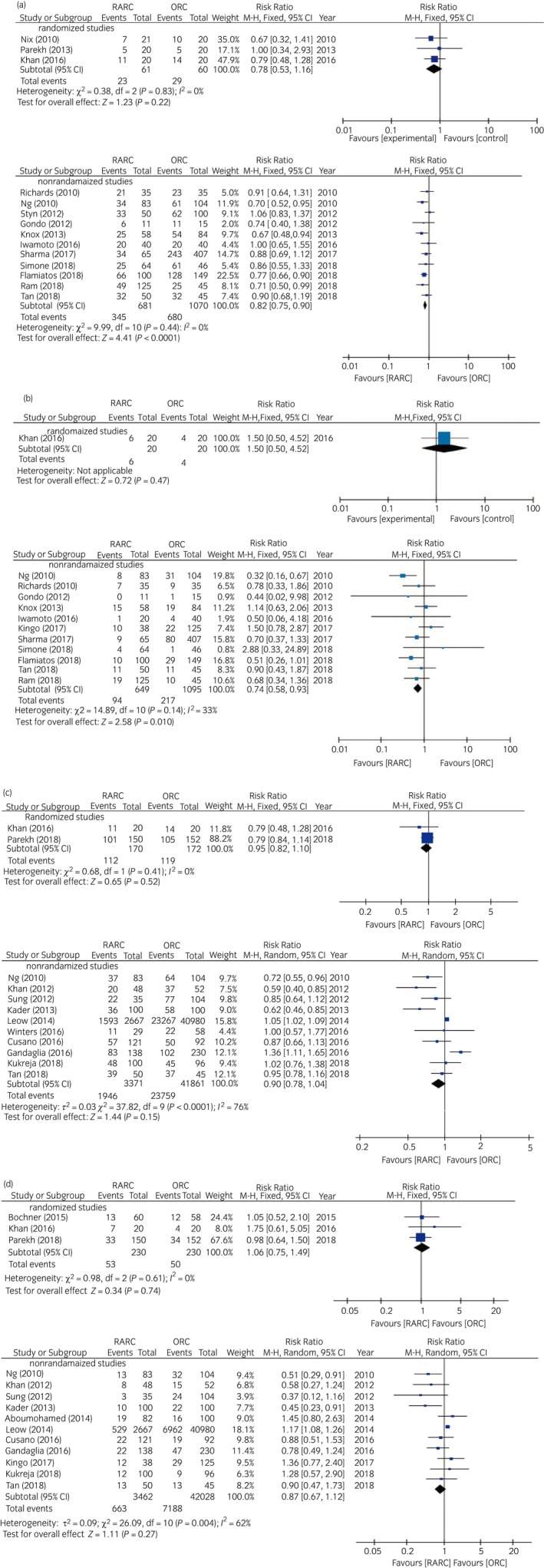
Forest plots showing the comparison of (a) rates for any grade of complication within 30 days, (b) rates for grade ≥3 of complication within 30 days, (c) rates for any grade of complication within 90 days and (d) rates for grade ≥3 of complication within 90 days.

Within the first 30 postoperative days, there was no significant difference in complications with any Clavien grade and Clavien grade ≥3 (RR 0.78, 95% CI 0.53–1.16, *P* = 0.22 and RR 1.50, 95% CI 0.50–4.52, *P* = 0.47, respectively) between RARC and ORC in RCTs (Fig. 3a,b). Conversely, RARC was associated with lower complication rates with any Clavien grade (RR 0.82, 95% CI 0.75–0.90, *P* < 0.0001) and Clavien grade ≥3 (RR 0.74, 95% CI 0.58–0.93, *P* = 0.01) in NRCTs (Fig. 3a,b). The χ^2^‐test and *I* ^2^‐test did not show any heterogeneity in all pooled analyses (Fig. 3a,b).

Within the first 90 postoperative days, there was no significant difference between RARC and ORC in complications with any Clavien grade and Clavien grade ≥3 both in RCTs (RR 0.95, 95% CI 0.82–1.10, *P* = 0.52 and RR 1.06, 95% CI 0.75–1.49, *P* = 0.74, respectively) and NRCTs (RR 0.90, 95% CI 0.78–1.04, *P* = 0.15 and RR 0.87, 95% CI 0.67–1.12, *P* = 0.27, respectively; Fig. 3c,d). The χ^2^‐test and *I* ^2^‐test showed significant heterogeneity in pooled analyses of NRCTs (Fig. 3c,d).

#### Comparison of mortality within 90 days between RARC and ORC

In RCTs, there was no significant difference between RARC and ORC in mortality rates within 90 days (RR 0.82, 95% CI 0.24–2.81, *P* = 0.75). Conversely, in NRCTs, RARC was associated with lower mortality rates within 90 days (RR 0.68, 95% CI 0.54–0.85, *P* = 0.0007; Fig. S2). The χ^2^‐test and *I* ^2^‐test did not show any heterogeneity in all pooled analyses (Fig. S2).

#### Subgroup analyses of types of complications between RARC and ORC

In RCTs, RARC was associated with higher overall UTI rates (RR 1.46, 95% CI 1.05–2.03, *P* = 0.03) compared with ORC without heterogeneity (Fig. S3a). In NRCTs, ORC was associated with higher overall thrombolic event rates (RR 0.42, 95% CI 0.21–0.82, *P* = 0.01) compared with RARC without heterogeneity (Fig. S3b). Figure S3c showed that there was no significant difference in ileus rates between RARC and ORC in both RCTs and NRCTs (RR 0.89, 95% CI 0.60–1.32, *P* = 0.56 and RR 0.89, 95% CI 0.63–1.25, *P* = 0.49, respectively).

#### Comparison of postoperative health‐related QOL between RARC and ORC

Three RCTs investigated postoperative health‐related QOL (Table 4); two studies used the FACT‐VCI and one study used the European Organization for Research and Treatment of Cancer QLQ‐C30. Messer *et al*. compared changes in health‐related QOL score between the RARC and ORC patients at baseline, 3, 6, 9 and 12 months after surgery using the FACT‐VCI in 40 patients.^45^ They compared deviation from preoperative baseline health‐related QOL, and concluded there was no significant difference between RARC and ORC in FACT‐VCI scores (RARC/ORC median: 119/135 at baseline, 127/136 at 3 months, 122/126 at 6 months, 142/128 at 9 months and 116/129 at 12 months) with the exception of a 2.5‐point lower score in the ORC arm for physical well‐being at 6 months by multivariable linear regression analysis.

Parekh *et al*. used FACT‐VCI in 302 patients to compare health‐related QOL at baseline with those at 3 and 6 months postoperatively.^2^ They found no significant difference between the RARC and ORC arms at any time point for total FACT‐VCI sores including all domains (RARC/ORC mean total: 120/121 at baseline, 123/125 at 3 months and 126/128 at 6 months). They also reported that mean total FACT‐VCI scores at 6 months postoperatively (RARC/ORC 126/128) in both arms had significantly improved after surgery compared with those at baseline (RARC/ORC 120/121).

Bochner *et al*. compared health‐related QOL at baseline with those at 3 and 6 months postoperatively in RARC and ORC using QLQ‐C30.^4^ They showed that there were no statistical differences in QLQ‐C30 change from baseline to 3 and 6 months after surgery in any of the evaluated domains.

In NRCTs (Table 4), Aboumohamed *et al*. compared health‐related QOL between the RARC and ORC in 182 patients preoperatively, and at 1, 3, 6, 12, 18, 24 and 30 months postoperatively utilizing BCI and the European Organization for Research and Treatment of Cancer BIS questionnaires.^25^ They reported that the BCI and BIS scores were not statistically different between the RARC and ORC arms at any time point for urinary function (*P* = 0.11), bowel function (*P* = 0.58) and body image (*P* = 0.93), but the sexual function at all time points was better for patients treated with ORC (*P* = 0.047). Conversely, Li *et al*. used the BCI and CARE tools to compare health‐related QOL between RARC and ORC in 324 patients at baseline, 1, 3, 6 and 12 months after surgery.^34^ They found no differences in any of the BCI domains (urinary, sexual and bowel domains) at any time point between both procedures.

## Discussion

Bladder cancer carries, due to the advanced age and high smoking rate of most patients, significant cumulative morbidity. RC, the standard of care for patients with muscle‐invasive and very high‐risk bladder cancer, is associated with a high rate of complications due to the complexity of the surgery itself and the inherent frailty of the patients.^36^ Minimal invasive surgeries, such as RARC, promise to reduce postoperative complications and maintain patients’ health‐related QOL despite its higher cost and need for a learning curve.^46^ While we are awaiting more robust data on the oncological equivalence of RARC to ORC, its benefits regarding postoperative complication rates and health‐related QOL are intensively debated. In a meta‐analysis published in 2015, Novara *et al*. reported that the rates for any and major (≥3) Clavien grade of complications within 90 days were slightly in favor of RARC.^47^ In this updated systematic review and meta‐analysis, our aim was to examine the most up‐to‐date data on the differential impact of these two procedures on perioperative complications, mortality and health‐related QOL, with a focus on the difference between the results of RCTs and NRCTs. Although NRCTs are known to potentially lead to a more biased evaluation and reporting of outcomes compared with RCTs, they are often the basis of decision‐making and guideline recommendations, especially in surgical specialties.

In this systematic review and meta‐analysis, we analyzed six RCTs and 31 NRCTs comprising 581 and 48 392 patients, respectively, treated with RARC or ORC. Additionally, we carried out meta‐analysis of RCTs and NRCTs separately to ensure bias‐sensitive analyses. We found that compared with ORC, RARC was consistently associated with lower estimated blood loss, lower transfusion rates and longer operative times in both RCTs and NRCTs. For example, the largest RCT^2^ and the largest NRCT^22^ both reported that RARC had significantly lower estimated blood loss, lower transfusion rates and longer operative times compared with ORC. This is consistent with the benefit of robotic surgery for other disease entities, such as robotic radical prostatectomy.^48^ Another potential benefit of robotic surgery has been the shortened length of stay. However, we did not find any difference in the length of stay between RARC and ORC in RCTs, whereas NRCTs did report a shorter length of stay. This might be due to changes in postoperative pathways and the learning curve with a benefit to more modern pathways, such as ERAS, which is known to shorten the length of stay.^8^ Indeed, in many NRCTs, RARC was carried out in a more recent time period than ORC, leading to an experience and practice pattern change bias favoring the more recent technique.

We found that there was no difference in postoperative complications and mortality between RARC and ORC. Interestingly, this was true for complications within 30 and 90 days, and mortality within 90 days in RCTs. However, in NRCTs, the mortality rate within 90 days and the complication rate within 30 days were worse for ORC. This reporting deviation from NRCTs could be due to a selection, reporting and/or detection bias in favor of RARC. For example, several studies included ORC patients with higher comorbidities, higher BMI and/or more advanced disease compared with those treated with RARC, which led to a selection bias benefiting RARC.^7^, ^16^, ^18^, ^19^, ^26^, ^33^, ^37^ Furthermore, when comparing novel interventions with conventional ones, there can be a novelty bias in NRCTs, which is a form of selection bias.^49^

There was no significant difference between RARC and ORC in the overall ileus rate in both RCTs and NRCTs. Additionally, postoperative health‐related QOL was not significantly different between RARC and ORC in both RCTs and NRCTs.

There are several limitations to be considered in this systematic review and meta‐analyses. There were just six RCTs included with a low number of patients, especially compared with NRCTs. However, all NRCTs were observational and retrospective, which can introduce selection bias. The operative time in RARC was reported including setting and console time in almost all eligible studies, therefore, we were not able to compare the exact operative time between console time and ORC. The definition of complications is slightly different among studies or not reported in several studies. The health‐related QOL assessment tools were very different between eligible studies. A surgeon's experience and expertise, which are only partially controlled in RCTs, were very different between studies, which might introduce a confounder.

Another limitation is the failure to report and control for the type and approach to the urinary diversion. Due to the heterogeneity regarding urinary diversion among eligible studies, we were not able to compare based on the type of urinary diversions. Furthermore, most urinary diversions described in RCTs and NRCTs of this meta‐analysis were carried out using ECUD, which might limit the benefits of RARC compared with ORC. Indeed, ICUD seems to have advantages in the postoperative complication rates and health‐related QOL after RARC compared with ECUD. Recently, in a retrospective study, Tan *et al*. showed the beneficial impact of RARC with ICUD using enhanced recovery protocols resulting in improved postoperative complication rates compared with ORC.^8^ However, this procedure requires a complicated surgical technique, which is only learned through a long learning curve. At this time, there is no prospective study to compare RARC with ICUD to RARC with ECUD or ORC.

## Conclusions

RARC leads to less blood loss and lower transfusion rates compared with ORC; ORC is, in turn, associated with shorter operative times. Our systematic review and meta‐analyses did not show any difference between RARC and ORC in postoperative complications, mortality, and health‐related QOL in RCTs. NRCTs consistently reported better perioperative outcomes for RARC, such as shorter length of stay, mortality rate within 90 days and complication rate within 30 days, which were not confirmed in RCTs. These discrepancies could be due to bias in study design, measurement, selection and reporting. Further RCTs comparing postoperative complications and health‐related QOL between RARC with ICUD and ORC are required to assess potential benefits of RARC with the varied forms of urinary diversion.

## Conflict of interest

None declared.

## Supporting information


**Figure S1.** Risk of bias table for RCTs.

**Figure S2.** Forest plot showing the comparison of mortality rates within 90 days between RARC and ORC.

**Figure S3.** Forest plots showing the comparison of (a) rates for UTI, (b) rates for thromboembolisis, and (c) rates for ileus between RARC and ORC.

**Table S1.** PRISMA 2009 checklist.

**Table S2.** Risk of bias assessment for NRCTs (ROBINS‐I).

## References

[1] Alfred Witjes J , Lebret T , Comperat EM *et al* Updated 2016 EAU guidelines on muscle‐invasive and metastatic bladder cancer. Eur. Urol. 2017; 71: 462–75. 27375033 10.1016/j.eururo.2016.06.020

[2] Parekh DJ , Reis IM , Castle EP *et al* Robot‐assisted radical cystectomy versus open radical cystectomy in patients with bladder cancer (RAZOR): an open‐label, randomised, phase 3, non‐inferiority trial. Lancet 2018; 391: 2525–36. 29976469 10.1016/S0140-6736(18)30996-6

[3] Rhee JJ , Lebeau S , Smolkin M , Theodorescu D . Radical cystectomy with ileal conduit diversion: early prospective evaluation of the impact of robotic assistance. BJU Int. 2006; 98: 1059–63. 16796697 10.1111/j.1464-410X.2006.06372.x

[4] Bochner BH , Dalbagni G , Sjoberg DD *et al* Comparing open radical cystectomy and robot‐assisted laparoscopic radical cystectomy: a randomized clinical trial. Eur. Urol. 2015; 67: 1042–50. 25496767 10.1016/j.eururo.2014.11.043 PMC4424172

[5] Khan MS , Gan C , Ahmed K *et al* A single‐centre early phase randomised controlled three‐arm trial of open, robotic, and laparoscopic radical cystectomy (CORAL). Eur. Urol. 2016; 69: 613–21. 26272237 10.1016/j.eururo.2015.07.038

[6] Lauridsen SV , Tonnesen H , Jensen BT , Neuner B , Thind P , Thomsen T . Complications and health‐related quality of life after robot‐assisted versus open radical cystectomy: a systematic review and meta‐analysis of four RCTs. Syst. Rev. 2017; 6: 150. 28768530 10.1186/s13643-017-0547-y PMC5541663

[7] Kukreja JB , Metcalfe MJ , Qiao W , Kamat AM , Dinney CPN , Navai N . Cost‐effectiveness of robot‐assisted radical cystectomy using a propensity‐matched cohort. Eur. Urol. Focus 2018; 10.1016/j.euf.2018.07.001. 30033071

[8] Tan WS , Tan M‐Y , Lamb BW *et al* Intracorporeal robot‐assisted radical cystectomy, together with an enhanced recovery programme, improves postoperative outcomes by aggregating marginal gains. BJU Int. 2018; 121: 632–9. 29124853 10.1111/bju.14073

[9] Liberati A , Altman DG , Tetzlaff J *et al* The PRISMA statement for reporting systematic reviews and meta‐analyses of studies that evaluate health care interventions: explanation and elaboration. J. Clin. Epidemiol. 2009; 62: e1–34. 19631507 10.1016/j.jclinepi.2009.06.006

[10] Higgins JP . Cochrane Handbook for Systematic Reviews of Intervensions. Version 5.1.0. (Updated March 2011). London: The Cochrane Collaboration, 2011.

[11] Hozo SP , Djulbegovic B , Hozo I . Estimating the mean and variance from the median, range, and the size of a sample. BMC Med. Res. Methodol. 2005; 5: 13. 15840177 10.1186/1471-2288-5-13 PMC1097734

[12] Sterne JA , Hernan MA , Reeves BC *et al* ROBINS‐I: a tool for assessing risk of bias in non‐randomised studies of interventions. BMJ 2016; 355: i4919. 27733354 10.1136/bmj.i4919 PMC5062054

[13] Nix J , Smith A , Kurpad R , Nielsen ME , Wallen EM , Pruthi RS . Prospective randomized controlled trial of robotic versus open radical cystectomy for bladder cancer: perioperative and pathologic results. Eur. Urol. 2010; 57: 196–201. 19853987 10.1016/j.eururo.2009.10.024

[14] Parekh DJ , Messer J , Fitzgerald J , Ercole B , Svatek R . Perioperative outcomes and oncologic efficacy from a pilot prospective randomized clinical trial of open versus robotic assisted radical cystectomy. J. Urol. 2013; 189: 474–9. 23017529 10.1016/j.juro.2012.09.077

[15] Messer JC , Punnen S , Fitzgerald J , Svatek R , Parekh DJ . Health‐related quality of life from a prospective randomised clinical trial of robot‐assisted laparoscopic vs open radical cystectomy. BJU Int. 2014; 114: 896–902. 24862633 10.1111/bju.12818

[16] Ng CK , Kauffman EC , Lee MM *et al* A comparison of postoperative complications in open versus robotic cystectomy. Eur. Urol. 2010; 57: 274–81. 19560255 10.1016/j.eururo.2009.06.001

[17] Richards KA , Hemal AK , Kader AK , Pettus JA . Robot assisted laparoscopic pelvic lymphadenectomy at the time of radical cystectomy rivals that of open surgery: single institution report. Urology 2010; 76: 1400–4. 20350755 10.1016/j.urology.2010.01.019

[18] Gondo T , Yoshioka K , Nakagami Y *et al* Robotic versus open radical cystectomy: prospective comparison of perioperative and pathologic outcomes in Japan. Jpn. J. Clin. Oncol. 2012; 42: 625–31. 22581913 10.1093/jjco/hys062

[19] Khan MS , Challacombe B , Elhage O *et al* A dual‐centre, cohort comparison of open, laparoscopic and robotic‐assisted radical cystectomy. Int. J. Clin. Pract. 2012; 66: 656–62. 22507234 10.1111/j.1742-1241.2011.02888.x

[20] Styn NR , Montgomery JS , Wood DP *et al* Matched comparison of robotic‐assisted and open radical cystectomy. Urology 2012; 79: 1303–8. 22516354 10.1016/j.urology.2012.01.055

[21] Sung HH , Ahn JS , Seo SI *et al* A comparison of early complications between open and robot‐assisted radical cystectomy. J. Endourol. 2012; 26: 670–5. 22011001 10.1089/end.2011.0372

[22] Kader AK , Richards KA , Krane LS , Pettus JA , Smith JJ , Hemal AK . Robot‐assisted laparoscopic vs open radical cystectomy: comparison of complications and perioperative oncological outcomes in 200 patients. BJU Int. 2013; 112: E290–4. 23815802 10.1111/bju.12167

[23] Knox ML , El‐Galley R , Busby JE . Robotic versus open radical cystectomy: identification of patients who benefit from the robotic approach. J. Endourol. 2013; 27: 40–4. 22788707 10.1089/end.2012.0168

[24] Maes AA , Brunkhorst LW , Gavin PW , Todd SP , Maatman TJ . Comparison of robotic‐assisted and open radical cystectomy in a community‐based, non‐tertiary health care setting. J. Robot. Surg. 2013; 7: 359–63. 27001875 10.1007/s11701-013-0401-8

[25] Aboumohamed AA , Raza SJ , Al‐Daghmin A *et al* Health‐related quality of life outcomes after robot‐assisted and open radical cystectomy using a validated bladder‐specific instrument: a multi‐institutional study. Urology 2014; 83: 1300–8. 24746661 10.1016/j.urology.2014.02.024

[26] Leow JJ , Reese SW , Jiang W *et al* Propensity‐matched comparison of morbidity and costs of open and robot‐assisted radical cystectomies: a contemporary population‐based analysis in the United States. Eur. Urol. 2014; 66: 569–76. 24491306 10.1016/j.eururo.2014.01.029

[27] Musch M , Janowski M , Steves A *et al* Comparison of early postoperative morbidity after robot‐assisted and open radical cystectomy: results of a prospective observational study. BJU Int. 2014; 113: 458–67. 24053793 10.1111/bju.12374

[28] Niegisch G , Albers P , Rabenalt R . Perioperative complications and oncological safety of robot‐assisted (RARC) vs. open radical cystectomy (ORC). Urol. Oncol. 2014; 32: 966–74. 25017695 10.1016/j.urolonc.2014.03.023

[29] Koupparis A , Villeda‐Sandoval C , Weale N , El‐Mahdy M , Gillatt D , Rowe E . Robot‐assisted radical cystectomy with intracorporeal urinary diversion: impact on an established enhanced recovery protocol. BJU Int. 2015; 116: 924–31. 25943158 10.1111/bju.13171

[30] Bak DJ , Lee YJ , Woo MJ *et al* Complications and oncologic outcomes following robot‐assisted radical cystectomy: what is the real benefit? Investig. Clin. Urol. 2016; 57: 260–7. 10.4111/icu.2016.57.4.260 PMC4949693 27437535

[31] Cusano A , Haddock P Jr , Jackson M , Staff I , Wagner J , Meraney A . A comparison of preliminary oncologic outcome and postoperative complications between patients undergoing either open or robotic radical cystectomy. Int. Braz. J. Urol. 2016; 42: 663–70. 27564275 10.1590/S1677-5538.IBJU.2015.0393 PMC5006760

[32] Gandaglia G , Karl A , Novara G *et al* Perioperative and oncologic outcomes of robot‐assisted vs. open radical cystectomy in bladder cancer patients: a comparison of two high‐volume referral centers. Eur. J. Surg. Oncol. 2016; 42: 1736–43. 27032295 10.1016/j.ejso.2016.02.254

[33] Iwamoto H , Yumioka T , Yamaguchi N *et al* Robot‐assisted radical cystectomy is a promising alternative to open surgery in the Japanese population with a high rate of octogenarians. Int. J. Clin. Oncol. 2016; 21: 756–63. 26792433 10.1007/s10147-016-0950-8

[34] Li AY , Filson CP , Hollingsworth JM *et al* Patient‐reported convalescence and quality of life recovery: a comparison of open and robotic‐assisted radical cystectomy. Surg. Innov. 2016; 23: 598–605. 27354552 10.1177/1553350616656284

[35] Satkunasivam R , Santomauro M , Chopra S *et al* Robotic intracorporeal orthotopic neobladder: urodynamic outcomes, urinary function, and health‐related quality of life. Eur. Urol. 2016; 69: 247–53. 26164417 10.1016/j.eururo.2015.06.041 PMC9083831

[36] Winters BR , Bremjit PJ , Gore JL *et al* Preliminary comparative effectiveness of robotic versus open radical cystectomy in elderly patients. J. Endourol. 2016; 30: 212–7. 26414964 10.1089/end.2015.0457

[37] Kingo PS , Norregaard R , Borre M , Jensen JB . Postoperative C‐reactive protein concentration and clinical outcome: comparison of open cystectomy to robot‐assisted laparoscopic cystectomy with extracorporeal or intracorporeal urinary diversion in a prospective study. Scand. J. Urol. 2017; 51: 381–7. 28678652 10.1080/21681805.2017.1334698

[38] Koie T , Ohyama C , Yamamoto H *et al* The feasibility and effectiveness of robot‐assisted radical cystectomy after neoadjuvant chemotherapy in patients with muscle‐invasive bladder cancer. Jpn. J. Clin. Oncol. 2017; 47: 252–6. 27980085 10.1093/jjco/hyw191

[39] Muto S , Kitamura K , Ieda T *et al* A preliminary oncologic outcome and postoperative complications in patients undergoing robot‐assisted radical cystectomy: initial experience. Investig. Clin. Urol. 2017; 58: 171–8. 10.4111/icu.2017.58.3.171 PMC5419105 28480342

[40] Sharma P , Zargar‐Shoshtari K , Poch MA *et al* Surgical control and margin status after robotic and open cystectomy in high‐risk cases: caution or equivalence? World J. Urol. 2017; 35: 657–63. 27495912 10.1007/s00345-016-1915-2

[41] Flamiatos JF , Chen Y , Lambert WE *et al* Open versus robot‐assisted radical cystectomy: 30‐day perioperative comparison and predictors for cost‐to‐patient, complication, and readmission. J. Robot. Surg. 2019; 13: 129–40. 29948875 10.1007/s11701-018-0832-3

[42] Panwar P , Mavuduru RS , Mete UK *et al* Perioperative outcomes of minimally invasive versus open radical cystectomy: a single‐center experience. Indian J. Urol. 2018; 34: 115–21. 29692504 10.4103/iju.IJU_166_17 PMC5894283

[43] Ram D , Rajappa SK , Rawal S , Singh A , Singh PB , Dewan AK . Is robot‐assisted radical cystectomy superior to standard open radical cystectomy? An Indian perspective J. Minim. Access Surg. 2018; 14: 298–303. 29483372 10.4103/jmas.JMAS_150_17 PMC6130186

[44] Simone G , Tuderti G , Misuraca L *et al* Perioperative and mid‐term oncologic outcomes of robotic assisted radical cystectomy with totally intracorporeal neobladder: results of a propensity score matched comparison with open cohort from a single‐centre series. Eur. J. Surg. Oncol. 2018; 44: 1432–8. 29699838 10.1016/j.ejso.2018.04.006

[45] Messer JC , Punnen S , Fitzgerald J , Svatek R , Parekh DJ . Health‐related quality of life from a prospective randomised clinical trial of robot‐assisted laparoscopic vs open radical cystectomy. BJU Int. 2013; 114: 896–902. 10.1111/bju.12818 24862633

[46] Hayn MH , Hussain A , Mansour AM *et al* The learning curve of robot‐assisted radical cystectomy: results from the International Robotic Cystectomy Consortium. Eur. Urol. 2010; 58: 197–202. 20434830 10.1016/j.eururo.2010.04.024

[47] Novara G , Catto JW , Wilson T *et al* Systematic review and cumulative analysis of perioperative outcomes and complications after robot‐assisted radical cystectomy. Eur. Urol. 2015; 67: 376–401. 25560798 10.1016/j.eururo.2014.12.007

[48] Leow JJ , Chang SL , Meyer CP *et al* Robot‐assisted versus open radical prostatectomy: a contemporary analysis of an all‐payer discharge database. Eur. Urol. 2016; 70: 837–45. 26874806 10.1016/j.eururo.2016.01.044

[49] Catalogue of Bias Collaboration . Persaud N HC. Novelty Bias: Catalogue of Bias. [Cited 25 Dec 2018.] Available from https://catalogofbiasorg/biases/novelty-bias/.

