# Supplementary figures and images for: Comparison of perioperative complications and health‐related quality of life between robot‐assisted and open radical cystectomy: A systematic review and meta‐analysis
Source: Int J Urol. 2019 May 13;26(8):760–74. doi: 10.1111/iju.14005 (PMC6851708; doi:10.1111/iju.14005)

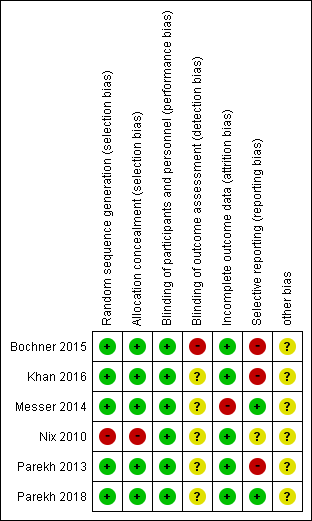


FigureS1: Risk of bias table for RCTs.

RCTs: randomized controlled studies

Supplement: Supplementary file 1 — Figure S1. Risk of bias table for RCTs. [file IJU-26-760-s001.docx]
