# Supplementary material for: Comparison of perioperative complications and health‐related quality of life between robot‐assisted and open radical cystectomy: A systematic review and meta‐analysis
Source: Int J Urol. 2019 May 13;26(8):760–74. doi: 10.1111/iju.14005 (PMC6851708; doi:10.1111/iju.14005)

FigureS2. Forest plots showing the comparison of mortality rates within 90days between RARC and ORC.

CI: confidence interval; M-H: Mantel-Haenszel test; ORC: open radical cystectomy; RARC: robot-assisted radical cystectomy; SD: standard deviation


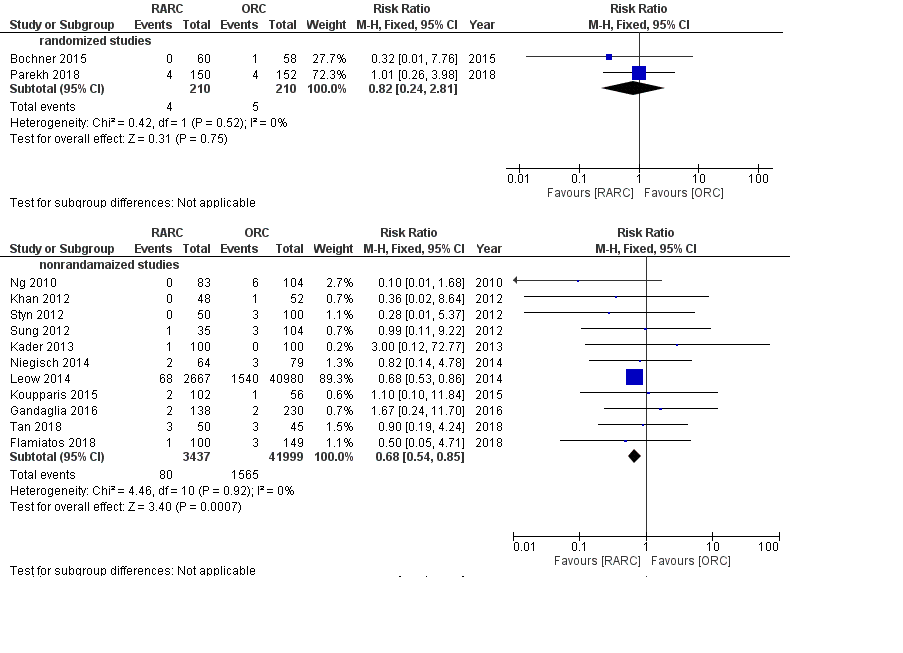

Supplement: Supplementary file 2 — Figure S2. Forest plot showing the comparison of mortality rates within 90 days between RARC and ORC. [file IJU-26-760-s002.docx]
