# Supplementary material for: Comparison of perioperative complications and health‐related quality of life between robot‐assisted and open radical cystectomy: A systematic review and meta‐analysis
Source: Int J Urol. 2019 May 13;26(8):760–74. doi: 10.1111/iju.14005 (PMC6851708; doi:10.1111/iju.14005)

FigureS3. Forest plots showing the comparison of (A) rates for UTI, (B) rates for thromboembolisis, (C) rates for ileus between RARC and ORC.

CI: confidence interval; M-H: Mantel-Haenszel test; ORC: open radical cystectomy; RARC: robot-assisted radical cystectomy; SD: standard deviation; UTI: urinary tract infection


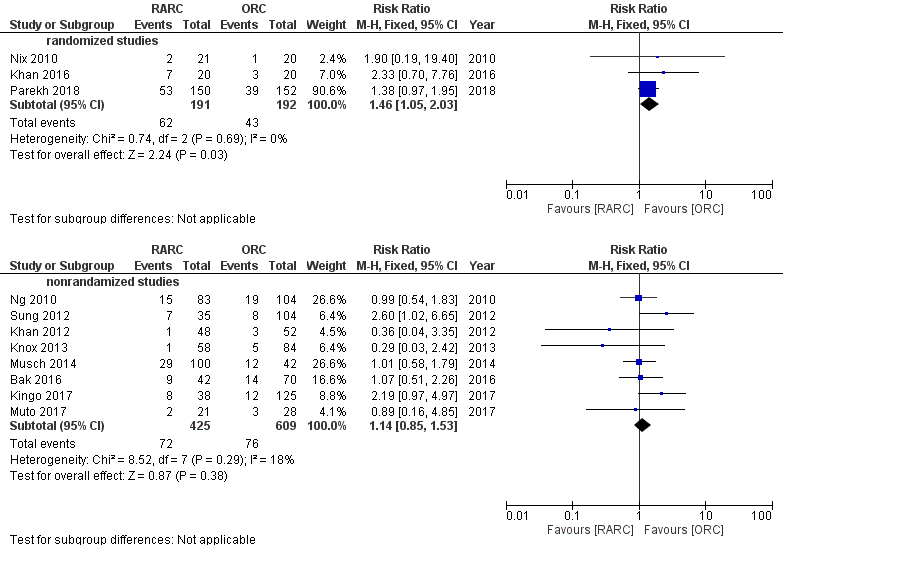


B.


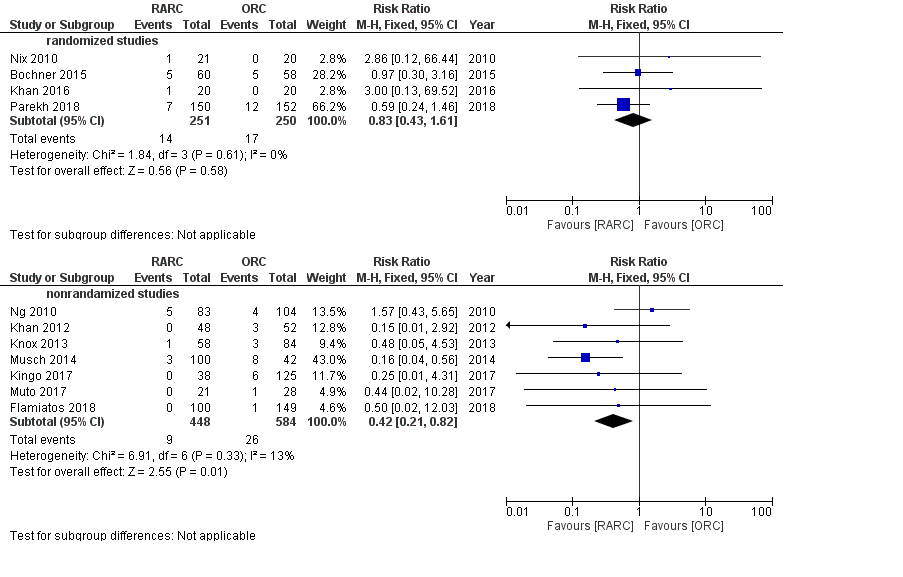


C.


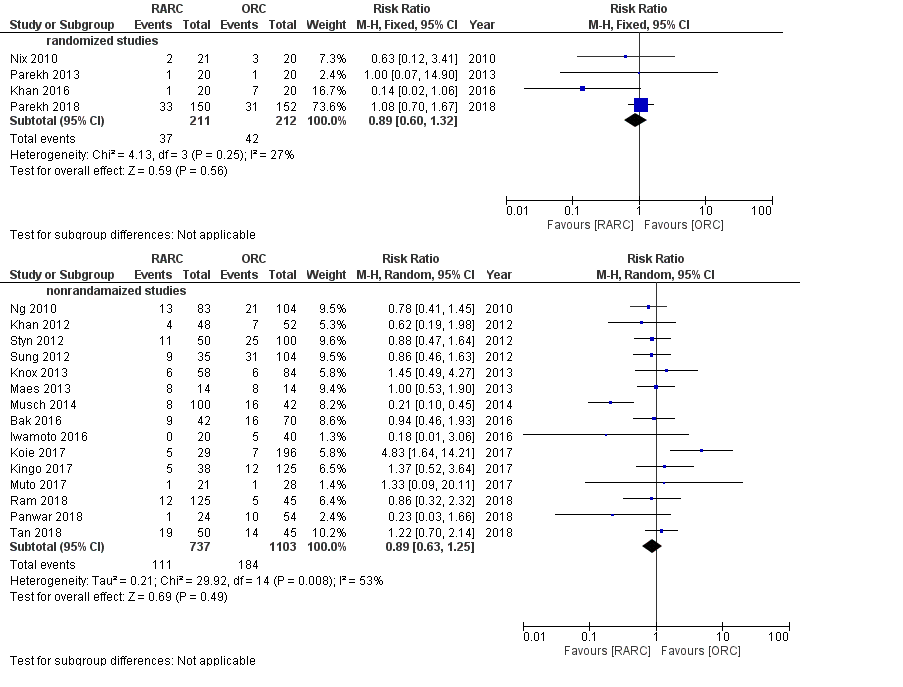

Supplement: Supplementary file 3 — Figure S3. Forest plots showing the comparison of (a) rates for UTI, (b) rates for thromboembolisis, and (c) rates for ileus between RARC and ORC. [file IJU-26-760-s003.docx]
