# Supplementary material for: Comparison of perioperative complications and health‐related quality of life between robot‐assisted and open radical cystectomy: A systematic review and meta‐analysis
Source: Int J Urol. 2019 May 13;26(8):760–74. doi: 10.1111/iju.14005 (PMC6851708; doi:10.1111/iju.14005)
Supplement: Supplementary file 5 — Table S2. Risk of bias assessment for NRCTs (ROBINS‐I). [file IJU-26-760-s005.docx]

| **TableS2.** Risk of bias assessment for NRCTs (ROBINS-I). | | | | | | | | |
| --- | --- | --- | --- | --- | --- | --- | --- | --- |
| **Author** | **Confounding** | **Participant selection** | **Classification of interventions** | **Deviations from intended intervention** | **Missing data** | **Measurement of outcomes** | **Selection of the**  **reported result** | **Overall** |
| Ng 2010 | Moderate | Moderate | Low | Moderate | Low | Low | Low | Moderate |
| Richards 2010 | Serious | Serious | Low | Serious | Low | Low | Moderate | Serious |
| Gondo 2012 | Serious | Serious | Low | Serious | Low | Low | Low | Serious |
| Khan 2012 | Moderate | Moderate | Low | Moderate | Low | Low | Low | Moderate |
| Styn 2012 | Serious | Serious | Low | Serious | Low | Low | Moderate | Serious |
| Sung 2012 | Moderate | Serious | Low | Serious | Low | Low | Moderate | Serious |
| Kander 2013 | Serious | Serious | Low | Serious | Low | Low | Moderate | Serious |
| Knox 2013 | Moderate | Moderate | Low | Moderate | Low | Low | Moderate | Moderate |
| Maes 2013 | Serious | Serious | Low | Low | Low | Low | Low | Serious |
| Aboumohamed 2014 | Serious | Serious | Low | Moderate | Moderate | Low | Moderate | Serious |
| Leow 2014 | Low | Low | Low | Low | Low | Low | Moderate | Moderate |
| Musch 2014 | Serious | Serious | Low | Serious | Serious | Low | Low | Serious |
| Niegisch 2014 | Moderate | Serious | Low | Serious | Low | Low | Moderate | Serious |
| Koupparis 2015 | Serious | Serious | Low | Serious | Low | Low | Moderate | Serious |
| Bak 2016 | Serious | Serious | Low | Serious | Low | Low | Moderate | Serious |
| Cusano 2016 | Serious | Serious | Low | Serious | Low | Low | Moderate | Serious |
| Gandaglia 2016 | Moderate | Moderate | Low | Serious | Low | Low | Moderate | Serious |
| Iwamoto 2016 | Moderate | Serious | Low | Serious | Low | Low | Moderate | Serious |
| Li 2016 | Moderate | Moderate | Low | Moderate | Moderate | Low | Serious | Serious |
| Satkunasivam 2016 | Moderate | Moderate | Low | Moderate | Moderate | Low | Serious | Serious |
| Winters 2016 | Serious | Serious | Low | Serious | Low | Low | Moderate | Serious |
| Kingo 2017 | Serious | Serious | Low | Serious | Low | Low | Low | Serious |
| Koie 2017 | Serious | Serious | Low | Serious | Low | Low | Moderate | Serious |
| Muto 2017 | Serious | Serious | Low | Serious | Low | Low | Moderate | Serious |
| Sharma 2017 | Moderate | Serious | Low | Serious | Low | Low | Low | Serious |
| Flamiatos 2018 | Moderate | Moderate | Low | Moderate | Low | Low | Moderate | Moderate |
| Kukreja 2018 | Moderate | Moderate | Low | Serious | Low | Low | Moderate | Serious |
| Simone 2018 | Moderate | Moderate | Low | Low | Low | Low | Moderate | Moderate |
| Tan 2018 | Moderate | Serious | Low | Serious | Low | Low | Moderate | Serious |
| Panwar 2018 | Serious | Serious | Low | Serious | Low | Low | Low | Serious |
| Ram 2018 | Serious | Serious | Low | Serious | Low | Low | Low | Serious |
| NRCTs: non-randomized comparative studies, ROBINS-I: Risk Of Bias In Non-Randomized Studies -of Interventions | | | | | | | | |
